# Language competency in autism: a scientometric review

**DOI:** 10.3389/fpsyt.2024.1338776

**Published:** 2024-03-25

**Authors:** Muhammad Alasmari, Ahmed Alduais, Fawaz Qasem

**Affiliations:** ^1^ Department of English Language and Literature, College of Letters and Arts, University of Bisha, Bisha, Saudi Arabia; ^2^ Department of Human Sciences (Psychology), University of Verona, Verona, Italy

**Keywords:** language acquisition, language development, autism spectrum disorder, scientometric review, language competence

## Abstract

The study of atypical language acquisition in children with, autism spectrum disorder (ASD) is crucial for both practical and theoretical reasons. Understanding the course of language development in ASD can inform potential interventions and treatments while shedding light on the necessary conditions for language development in typically developing children. This scientometric review aims to provide a comprehensive overview of the research landscape in this field, identifying trends, patterns, and knowledge gaps. The methods employed in this review comprise a systematic search of three major databases: Scopus (5,026 documents), Web of Science (WoS; 4,570 documents), and Lens (3,235 documents). The analysis includes bibliometric indicators such as knowledge production size by year, country, university, source, subject area, author, and citation. Scientometric indicators consist of burst detection, silhouette, clusters, citation, and co-occurrence of keywords. The analysis reveals clusters focusing on various aspects of language development in ASD, such as motor skills, parental communication strategies, cognitive processes, and genetics. Key clusters include the relationship between fine motor gestures and language usage patterns, the role of expressive language skills and maternal gesture use, and the effectiveness of online parent training modules for improving prelinguistic predictors. Other noteworthy clusters explore the importance of core language skills, the role of natural language input and syntactic complexity, and the genetic underpinnings of language abilities in high-functioning adults with ASD. In conclusion, this scientometric review highlights the top 10 clusters and their respective Silhouette values, providing valuable insights into language acquisition in ASD. These findings have important implications for guiding future research directions and informing the creation of targeted and effective interventions to support language acquisition in this population.

## 1 Introduction

The investigation into language development provides a window into the complex interplay between cognitive processes and communicative abilities, a subject that holds a central place within cognitive science. In studying this development, researchers gain invaluable insights into the developmental milestones achieved by typically developing children, as well as the unique challenges faced by those with language disorders, including those diagnosed with ASD. The linguistic journey of children with ASD is characterized by a distinct profile of verbal communication challenges, encompassing comprehension and production across various linguistic domains such as phonetics, phonology, syntax, and pragmatics. These atypical traits are underpinned by cognitive mechanisms that differ from typical language development, leading to a unique pattern of linguistic abilities (1, 2).

ASD is a condition identifiable by its onset in early childhood, typically between 12 to 14 months of age, and presents with distinctive challenges in social interaction, communication, and a tendency towards repetitive behaviours (3). The definition of ASD is dynamic, continuously evolving with advances in clinical diagnoses and a deeper understanding of the nature of autism-related syndromes. Traditionally viewed as a neurological and developmental disorder, ASD’s impact is profound on an individual’s ability to interact socially, process learning, and exhibit adaptive behaviour (3). Symptoms generally manifest during the first two years of a child’s life, with enduring impairments in social interaction and the presence of restricted, repetitive patterns of behaviour, interests, or activities as defining aspects of ASD (3).

The prevalence of ASD is not limited by geographic or linguistic boundaries, affecting diverse populations across the globe. Over several decades, there has been a significant upsurge in ASD diagnoses, a trend consistently reported on an international scale. An analysis of 61 studies from 18 countries, conducted from 1966 to 2009, revealed an overall prevalence of 0.7%, which equates to approximately 1 in 143 children (4). More recent data indicate an even greater prevalence, increasing from 6.7 per 1,000 children aged 8 years in 2000 and 2002, to 18.5 per 1,000 in 2016. This same study noted a decline in the proportion of children with ASD who also have an intellectual disability, dropping from about half in 2000 and 2002 to one-third by 2016 (5).

To diagnose and assess language disorders such as ASD, a variety of tools and methodologies are employed. Despite significant strides in improving diagnostic and assessment tools, the mean age at which ASD is diagnosed remains between 4 to 5 years. Widely used diagnostic instruments like the Autism Diagnostic Observational Schedule (ADOS) and the Communication and Symbolic Behaviour Scales (CSBS) have been lauded for their meticulous assessment capabilities, particularly in young children (6, 7).

The field of language acquisition in ASD is vibrant with research, offering new insights into caregiver interactions, early social communication, and even molecular genetics that influence language development. Caregiver engagement with a child’s utterances and the language environment at home are critical factors that affect language acquisition in children with ASD, as demonstrated by studies from Fusaroli et al. and Swanson (8, 9). These findings are reinforced by research indicating the importance of early language exposure and the influence of parental linguistic alignment on language development, with studies noting that maternal and paternal interactions may differ in their effects on child language skills (10, 11).

The importance of early social communication skills as predictors for later language development has been emphasized by researchers such as Pickles et al. and Swanson (12, 13). Furthermore, biological underpinnings of language development in ASD, such as the broad autism phenotype and genetic influences, have been examined to understand their impact on language abilities (14, 15). This line of research suggests a potential correlation between cognitive capacities and language development in individuals with ASD.

Moreover, the role of pragmatic language markers in the diagnosis and assessment of ASD severity has been explored, with a growing understanding of how pragmatics can enhance typical interactions and adapt to the surrounding environment (15, 16). Intervention strategies, such as verbal communication and music-mediated approaches, have been investigated for their potential to improve language skills in children with ASD, highlighting the promise of these techniques in supporting those with limited spoken language abilities (17). The exploration of language development within the realm of cognitive science offers profound insights into the complex processes underlying the acquisition of language, and it is particularly revealing when considering children with ASD. These children frequently exhibit atypical linguistic traits, which can manifest as difficulties in both the comprehension and production of language. The linguistic characteristics of this atypical development—including variations in phonetics, phonology, syntax, and pragmatics—are shaped by cognitive mechanisms that differ from typical developmental processes (1, 2).

Children with ASD, a condition emerging typically between 12 to 14 months of age, often face distinctive challenges in social interaction and communication, as well as a propensity for repetitive behaviours (3). The criteria defining ASD are in a state of constant evolution, reflecting the ongoing advancements in clinical understandings and diagnosis. The disorder predominantly affects an individual’s social interaction and behaviour, with symptoms usually becoming noticeable within the first two years of life. These include persistent impairments in social communication and the presence of restricted, repetitive patterns of behaviours, interests, or activities, which are considered hallmark features of ASD.

The incidence of ASD transcends geographical and linguistic boundaries, affecting populations worldwide. Studies have documented a marked increase in ASD diagnoses over recent decades. An extensive review of 61 studies across 18 countries, from 1966 to 2009, indicated an overall prevalence of 0.7%, or about 1 in 143 children (4). More recent statistics suggest an increase, with prevalence rates rising from 6.7 per 1,000 children aged 8 years in the early 2000s to 18.5 per 1,000 in 2016. This research also observed a decrease in the incidence of intellectual disability among children diagnosed with ASD, from roughly 50% in earlier studies to about 33% in 2016 (5).

The diagnosis and assessment of language disorders, including ASD, are reliant on a range of tools and methods. Despite significant advancements in the field, the average age at which ASD is diagnosed has remained between 4 to 5 years. Diagnostic instruments such as the Autism Diagnostic Observational Schedule (ADOS) and the Communication and Symbolic Behaviour Scales (CSBS) are commonly used due to their thorough assessment capabilities, especially in young children (6, 7).

Recent research into language acquisition in individuals with ASD has delved into various dimensions, such as the influence of caregiver interactions, early social communication skills, and genetic factors on language development. Studies by Fusaroli et al. and Swanson have underscored the significance of caregiver engagement and the language environment at home on the language development of children with ASD (8, 9). These findings align with other research pointing toward the importance of early language exposure and the effect of parental linguistic alignment on language skills development, with noted differences in maternal and paternal communication styles (10, 11). Equally important is the standardization of methodologies in language disorder research, which will enable more accurate comparisons and understandings of language skills in adults with NDDs, ultimately guiding the enhancement of targeted therapies and support systems (18).

Early social communication skills have been identified as predictors for later language development in children with ASD, as evidenced by the work of Pickles et al. and Swanson (12, 13). Additionally, the biological foundations of language development in ASD, such as genetic influences and the broad autism phenotype, have been investigated to discern their impact on linguistic abilities (15, 19). This line of inquiry suggests a linkage between cognitive abilities and language development in ASD.

The significance of pragmatic language markers in diagnosing ASD and determining its severity has also been a focus, with pragmatics playing an increasingly recognized role in understanding human interaction and helping children with ASD navigate their environment (15, 16). Research into intervention strategies, including verbal communication and music-mediated approaches, has shown potential for enhancing language skills in children with ASD, particularly for those with limited spoken language abilities (17, 20, 21).

Considering caregiver speech’s impact on language development, studies by Ravi et al. and McDaniel et al. have highlighted the significance of parental behaviour during the earliest stages of life on later developmental outcomes for children with ASD (11, 22). Furthermore, the work of Alduais et al. and Swanson et al. has brought attention to the importance of pragmatic language skills in communication, suggesting that tools such as the CCC-2 questionnaire could support clinicians in the autism diagnosis process (13, 23).

Identifying the gaps in research is essential for enhancing our understanding of language acquisition and development in children with ASD. While significant progress has been made, several areas necessitate further investigation. For instance, the impact of ASD severity on language development remains underexplored. Studies have provided inconsistent findings, indicating a need for more comprehensive research to understand how varying degrees of ASD affect language skills (24, 25).

Additionally, the benefits of longitudinal research cannot be overstated. Such studies would contribute to a more nuanced understanding of language development trajectories in children with ASD by following the same individuals over time. Although initial steps have been made in this direction (26), further long-term studies are critical to map out these trajectories in greater detail.

The potential of machine learning approaches in detecting ASD from linguistic samples is another burgeoning area of research. Initial studies harnessing technologies like ELMo and USE have shown promise in identifying ASD from narrative texts (27). Nevertheless, validating these findings across larger and more diverse samples is necessary to ensure the reliability and generalizability of these methods.

Lastly, the exploration of parental strategies for supporting language development in ASD has been a subject of considerable interest. While the importance of the home language environment and parental interaction has been established (9, 20), there is still a need to identify the most effective strategies and understand how these can be customized to cater to the individual needs of each child with ASD.

In summary, addressing these research gaps is crucial for the advancement of diagnostic tools, the development of effective interventions, and the improvement of language outcomes for children with ASD. As the field of ASD language acquisition research moves forward, a multi-faceted approach that includes a combination of genetic, environmental, and interactive factors will be necessary to fully support language development in these individuals. The integration of new methodologies, long-term studies, and personalized interventions will undoubtedly contribute to the growing body of knowledge, ultimately facilitating better support for children with ASD and their families.

### 1.1 Purpose of the present study

The aim of this study is to provide a comprehensive analysis of the scientific literature on language acquisition in ASD by employing scientometric methods to uncover trends, patterns, and knowledge gaps in the field. By examining factors such as publication frequency, citation analysis, research themes, and patterns, this review seeks to identify the most influential research, emerging areas of interest, and potential avenues for future investigation. This comprehensive overview of the language development research landscape in ASD aims to facilitate a better understanding of the current state of knowledge and to inform the development of future research strategies, ultimately contributing to the advancement of evidence-based practices and interventions for individuals with ASD and their families. The present study has been intentionally crafted with an expansive purview to scrutinize the overarching patterns currently steering scholarly endeavours in the realm of language studies, with a particular emphasis (but not limited to) language acquisition. While the intricacies of bilingualism and multilingualism undoubtedly warrant scholarly attention within this investigative sphere, we have elected to confine our analysis to the domain of first language acquisition. This decision was informed by the stronger association between the nuances of primary language learning and ASD, rather than a broader inquiry into language acquisition processes. Further, we have employed the terms ‘acquisition’ and ‘development’ in a synonymous manner throughout this review to denote the same conceptual understanding. Guiding our scientometric analysis, three research questions were meticulously formulated:

1. What are the primary trends in ASD language acquisition research, including top topics, methods, and publication geography?
2. Who are the key contributors to ASD language acquisition research in terms of publication quantity, citation impact, and collaboration, and what factors contribute to their success?
3. What are the emerging interests and potential knowledge gaps in ASD language acquisition research, and how can future studies address these gaps to enhance our knowledge and support for those with autism and their families?

## 2 Methods

### 2.1 Research methods

In its most basic form, scientometrics can be described as the analysis of scientific artifacts, focusing not on the science and scholarship itself but on the outcomes of these activities (28). The primary concern of researchers in this domain is to investigate the quantitative aspects of scientific information production, dissemination, and utilization, with the goal of gaining a deeper understanding of the mechanisms behind scientific research as a social activity (29). The scholarly discourse surrounding the efficacy of scientometric studies as a means for appraising the calibre of published research remains a contentious issue. This debate is particularly salient within the fields of bibliometrics, informetrics, and scientometrics, where a diverse range of researchers’ backgrounds can introduce a multiplicity of methodological perspectives and evaluative criteria, complicating the assessment of scholarly quality. As Egghe (30) has pointed out, the heterogeneity inherent in these fields poses distinct challenges for the objective evaluation of research outputs, suggesting that traditional metrics may not fully encapsulate the multifaceted nature of scholarly contributions.

Despite these challenges, the objectives of scientometric studies have consistently evolved to reflect the dynamics of scientific inquiry more accurately. Parkinson (31) emphasizes that the fundamental purpose of these investigations—to reveal and analyse the characteristics of scientometric phenomena and processes—remains crucial for the advancement of science management. This includes understanding the distribution of research, collaboration patterns, and the impact of studies within a given field. In selecting scientometric methods for our review of language acquisition within the context of ASD, we recognize the debate regarding its efficacy. However, we assert that scientometrics is justified due to its ability to offer a comprehensive and objective mapping of research trends, provide quantitative analysis, facilitate benchmarking against other fields, and synthesize interdisciplinary studies into a coherent overview. This methodological approach, despite its scrutinized limitations, affords a unique and valuable perspective on the evolution and impact of the field, which is essential for identifying areas for further investigation and for the effective management of scientific research. We commit to addressing the methodological concerns by incorporating a balanced discussion supported by current literature that acknowledges the complexities and critiques associated with scientometric evaluations.

Scientometric studies are guided by various indicators, which may be related to elements (e.g., publications, citations, references, potential) or types (e.g., quantitative, impact) (31). Another important aspect worth mentioning is “mapping knowledge domains”. This process involves creating a visual representation that illustrates the developmental progress and structural relationships of scientific knowledge, with maps serving as valuable tools for monitoring the frontiers of science and technology, enhancing knowledge management, and supporting scientific and technological decision-making (32). A recent study suggested that this approach could be applied across diverse fields of study, not just restricted to medical, health, and pure sciences (33). The current study investigates language acquisition in ASD.

### 2.2 Measures

Both bibliometric and scientometric research methods contribute to evaluating the knowledge generated within a particular domain (34, 35), such as language acquisition in ASD. Typically, bibliographic indicators can be found in knowledge databases like Scopus, WoS, and Lens (36–38). On the other hand, scientometric indicators are often supplied through specialized software tools. In our investigation, we utilized two such software applications: CiteSpace 5.8.R3 (39) and VOSviewer 1.6.18 (40). These programs facilitated the analysis and visualization of a variety of bibliometric and scientometric indicators, which are detailed in **Table 1**. The use of these software tools and databases allowed us to gain a more profound understanding of the knowledge creation, distribution, and influence in language acquisition and development in ASD. This understanding can help inform future research endeavours, pinpoint gaps in existing literature, and promote the development of more effective, targeted interventions within this interdisciplinary field.

**Table 1:** Bibliometric and scientometric indicators for measuring language acquisition in ASD.

Indicator	Definition/specification/retrieved data	Database/Software			
		Scopus	WOS		Lens
Bibliometric					
Year	Production size by year	√	√		√
Country	Top countries publishing in the field	√	√		√
University	Top universities, research centres, etc.	√	√		√
Source	Top journals, book series, etc.	√	√		√
Subject area	Top fields associated with the field	√	√		√
Author	Top authors publishing in the field	√	√		√
Citation	Top cited documents	√	√		√

Scientometric		CiteSpace	VOSviewer		
Burst detection	Determines the frequency of a certain event in certain period (e.g., the frequent citation of a certain reference during a period of time) [42]	√	×		
Silhouette	Used in cluster analysis to measure consistency of each cluster with its related nodes [41]	√	×		
Clusters	“We can probably eyeball the visualized network and identify some prominent groupings” [41] (p. 23).	√	√		
Citation	“The relatedness of items is determined based on the number of times they cite each other” [40] (p. 5). Units of analysis include documents, sources, authors, organizations, or countries.	√	√		
Keywords	CiteSpace provides co-occurring author keywords and keywords plus. In VOSviewer, co-occurrence analysis is defined as “the relatedness of items is determined based on the number of documents in which they occur together” [40] (p. 5). Units of analysis include author keywords, all keywords, or keywords plus.	√	√		

The Silhouette value is a measure of how similar an object is to its own cluster compared to other clusters. It ranges from -1 to +1, where a high value indicates that the object is well matched to its own cluster and poorly matched to neighbouring clusters. In our study, we used the Silhouette value as a metric to evaluate the quality of our clustering of the references, with higher Silhouette values indicating more relevant and closely related references within a particular cluster (41).

The use of Silhouette values in research allows for a quantitative measure of the quality of clustering, which can be particularly valuable in studies like ours where we are dealing with many references. By utilizing this measure, we can ensure our clusters are meaningful and relevant, thereby strengthening the findings and conclusions drawn from our cluster analysis (41).

### 2.3 Data-collection and sample

Data were collected from Scopus, WoS, and Lens for several justifiable reasons. Scopus and WoS are not only extensive knowledge databases containing a vast array of knowledge-based articles, but they also encompass a wide variety of source materials curated for their research quality (14, 42, 43). Furthermore, Lens has been recognized for its greater comprehensiveness compared to the other two databases, as it covers a broader range of data not incorporated in Scopus and WoS, making it a superior database overall (14, 44, 45).

The data search was conducted on Monday, 3 July 2023. We did not impose any language restrictions, provided that the title, abstract, and keywords were available in English. As a result, a manual verification process was implemented due to the scarcity of results in languages other than English. This study included all document types containing full-text, encompassing every kind of document with full-text availability. For more detailed information on the search strings employed in the three databases and additional specifications, please refer to **Table 2**. The table presents the number of articles found in three databases (Scopus, WoS, and Lens) based on two sets of search strings related to language acquisition and ASD. The ‘Detailed’ search strings focus on the title, abstract, and keywords and yield larger numbers of articles, while the ‘Specific’ search strings focus solely on the title, yielding fewer articles.

**Table 2:** Search strings for retrieving data on language acquisition in ASD from Scopus, WOS, and Lens.

Query	Search strings: Searched on 03 July 2023	Scopus	WoS	Lens
Detailed	(TITLE-ABS-KEY ("language development" OR "language acquisition" OR "language skills" OR "language ability" OR "language competence") AND TITLE-ABS-KEY ("autism" OR "autism spectrum" OR "autism spectrum disorder" OR "Autistic Disorder" OR "Asperger Disorder" OR "Pervasive Developmental Disorder Not Otherwise Specified" OR "Asperger's syndrome" OR "autistic" OR "high functioning autism" OR "low functioning autism" OR "Pervasive developmental disorder" OR "Childhood autism" OR "Atypical autism"))	5026	4570	3235
Specific	(TITLE ("language development" OR "language acquisition" OR "language skills" OR "language ability" OR "language competence" ) AND TITLE ("autism" OR "autism spectrum" OR "autism spectrum disorder" OR "Autistic Disorder" OR "Asperger Disorder" OR "Pervasive Developmental Disorder Not Otherwise Specified" OR "Asperger's syndrome" OR "autistic" OR "high functioning autism" OR "low functioning autism" OR "Pervasive developmental disorder" OR "Childhood autism" OR "Atypical autism"))	249	190	459

### 2.4 Data analysis

The data analysis procedure consisted of several stages, both before and after the analysis process.

1. Scopus data exported in three formats: Excel sheets, CiteSpace RIS files, and VOSviewer CSV files.
2. WoS data extracted in two formats: Excel sheets for bibliometric analysis and plain text documents for CiteSpace and VOSviewer.
3. Lens data obtained in two formats: CSV for bibliometric analysis and full record CSV for VOSviewer visualization.
4. Duplicates removed using CiteSpace and Mendeley.
5. Excel used for bibliometric analysis and generating citation reports.
6. Scientometric analysis settings set to default.
7. Two visualizations created on Lens: network and density.
8. The data were analysed for co-occurrences by keywords.
9. Scopus and WoS analysed in CiteSpace for and occurrences (keywords) and clusters.
10. Analysis results: charts, tables, narrative summaries, cluster summaries, visual maps, and burst tables.

Various document types were retained, with a majority being articles. Other document types included book chapters, early access articles, proceedings papers, book reviews, corrections, editorial materials, meeting abstracts, notes, and reviews. The analysis effectively used the remove duplicates feature in CiteSpace, ensuring the accuracy and reliability of the data.

## 3 Results

### 3.1 Trends of language acquisition in ASD

**Figure 1:**
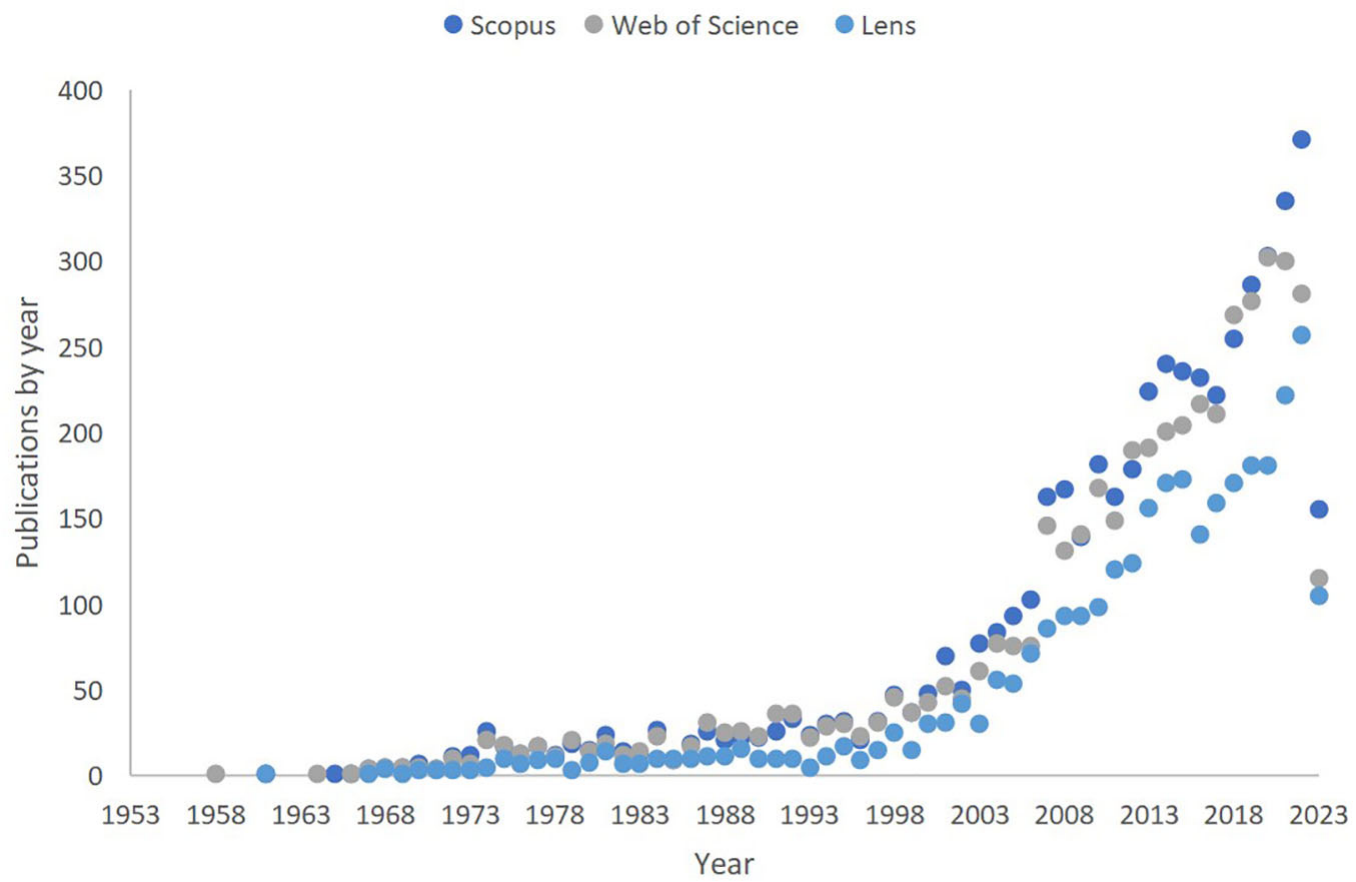
Annual Knowledge Production in Language Acquisition in ASD: A Comparison of Scopus, WoS, and Lens Data.

The data presented in **Figure 1** highlights the production of research knowledge on language acquisition in ASD over the years, as indexed in three major databases: Scopus, WoS, and Lens. A notable increase in the number of published documents on the topic can be observed across all three databases, reflecting a growing interest and investment in this research area. In Scopus, the number of publications saw a significant rise from a mere 1 in 1961 to 371 in 2022, while in WoS, the upward trend is evident from 1 publication in 1958 to 281 in 2022. Similarly, Lens shows an increase from 1 publication in 1961 to 257 in 2022. These trends suggest that the investigation of language acquisition in ASD has gained substantial attention in the scientific community over the years, and this momentum is likely to continue as researchers explore new methods, theories, and interventions to better understand and support individuals with autism.

### 3.2 Key contributors to language acquisition in ASD

**Figure 2:**
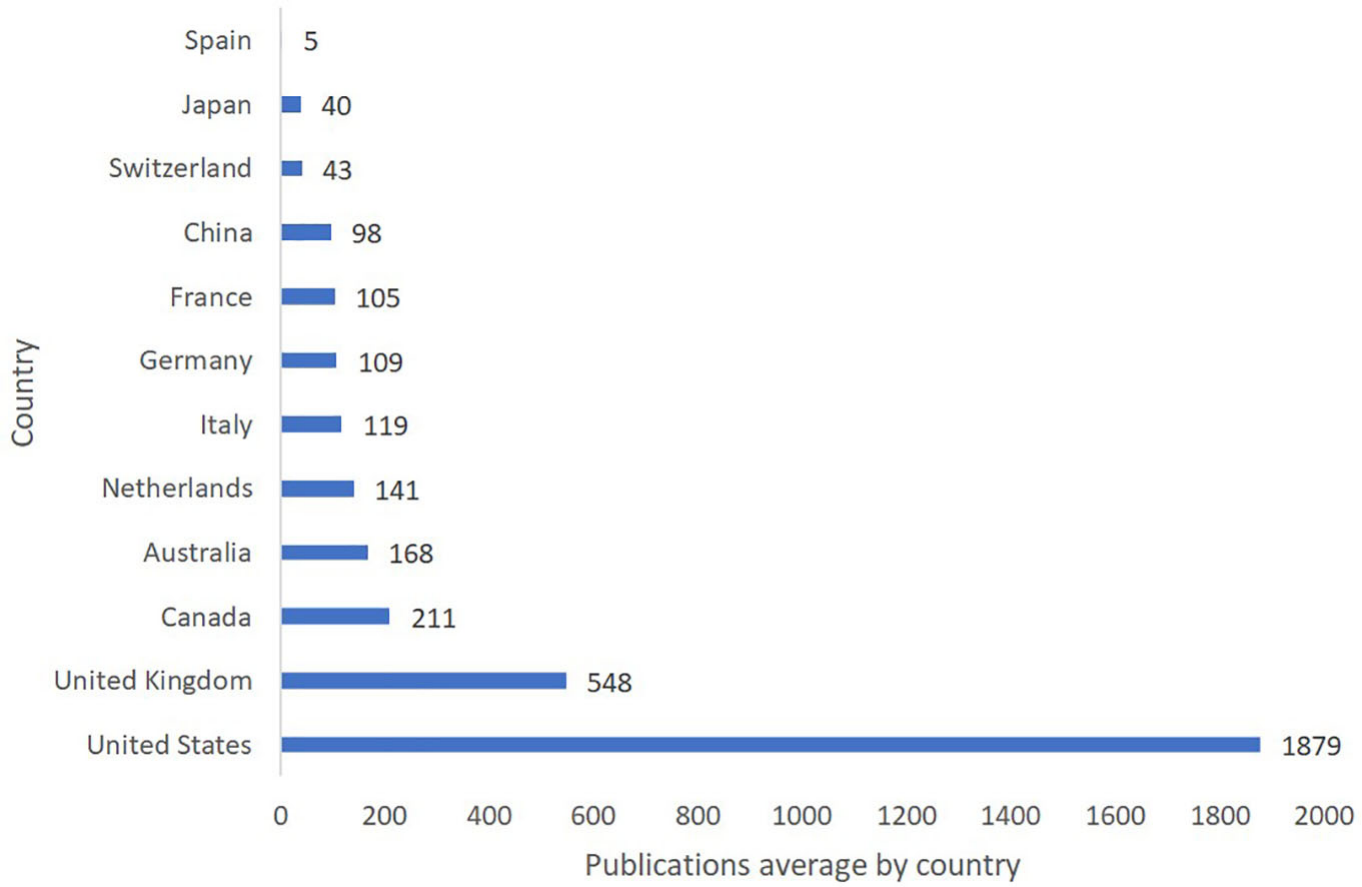
Language Acquisition in ASD: Knowledge Production Across Countries.

The data presented in **Figure 2** highlights the top publishing countries in the research area of language acquisition in ASD, based on the average number of publications across three databases: Scopus, WoS, and Lens. The United States leads the way with a total of 1879 publications, followed by the United Kingdom with 548 publications. Canada holds the third position with 211 publications. Australia, the Netherlands, Italy, Germany, France, and China have also contributed significantly to the field, with 168, 141, 119, 109, 105, and 98 publications, respectively. Switzerland and Japan follow with 43 and 40 publications. Spain, however, has an average of only 5 publications in this research area. This distribution of research production demonstrates that most of the knowledge on language acquisition in ASD is generated by countries with well-established research infrastructure and resources. It also underscores the importance of fostering international collaboration and knowledge sharing to advance our understanding of autism and language development across diverse cultural and geographic contexts.

**Figure 3:**
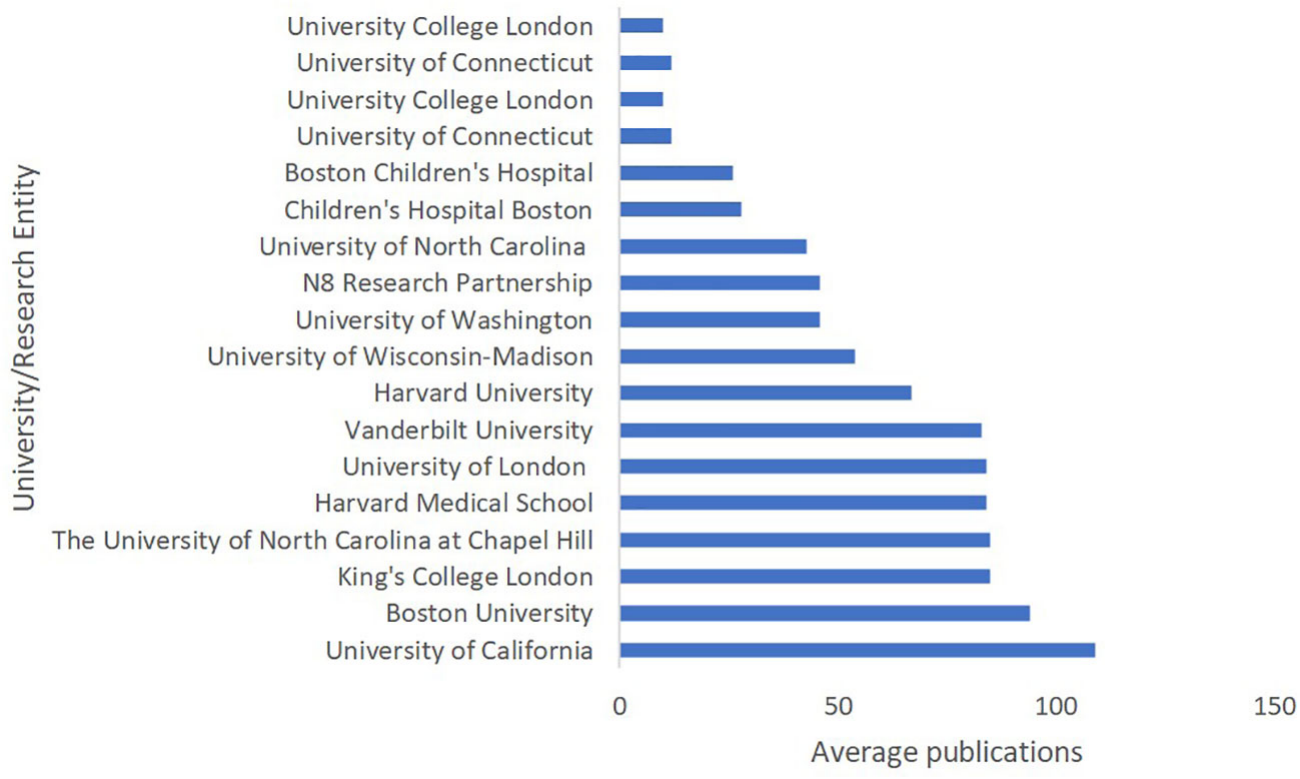
Language Acquisition in ASD: Knowledge Production by Affiliations.

The data presented in **Figure 3** provides an overview of the top publishing affiliations in the research area of Language Acquisition in ASD, based on the average number of publications across three databases: Scopus, WoS, and Lens. Leading the list is the University of California with 109 publications, followed closely by Boston University with 94 publications, and the University of California, Los Angeles with 92 publications. Other prominent institutions contributing to the field include King’s College London, The University of North Carolina at Chapel Hill, Harvard Medical School, University of London, and Vanderbilt University, with publication counts ranging from 85 to 83. Harvard University, University of Wisconsin-Madison, University of Washington, N8 Research Partnership, and University of California, Davis also make significant contributions, with publications ranging from 67 to 45. Institutions such as the University of North Carolina, Children’s Hospital Boston, Boston Children’s Hospital, University of Connecticut, and University College London have lower average publication counts, ranging from 43 to 10. This distribution of research production highlights the significant contributions made by prestigious academic institutions in advancing our understanding of language acquisition in ASD, emphasizing the importance of continued research and collaboration among these entities.

**Figure 4:**
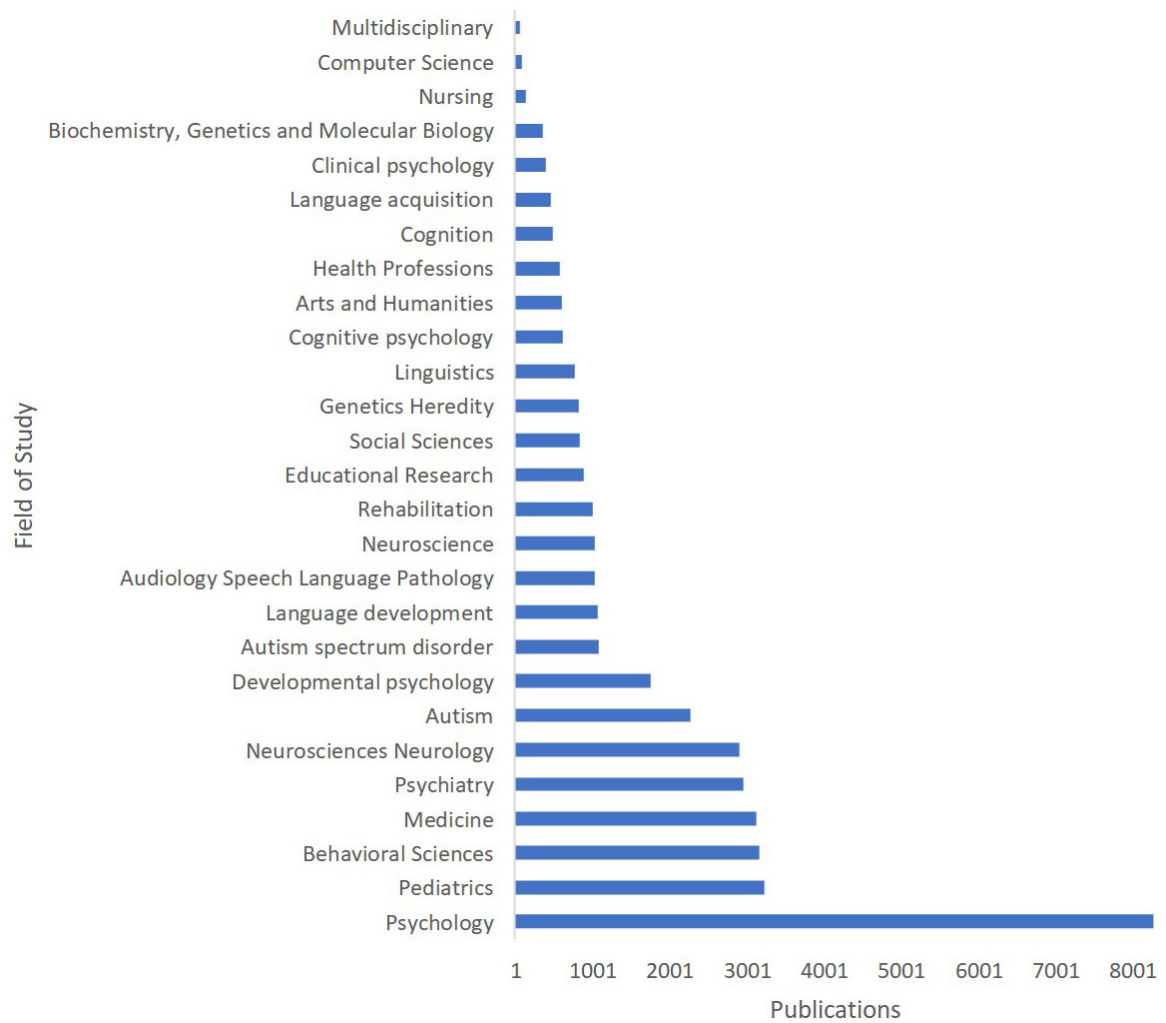
Language Acquisition in ASD: Knowledge Production by Field of Study.

The data presented in **Figure 4** showcases the production of knowledge on language acquisition in ASD across various fields of study within three databases: Scopus, WoS, and Lens. By grouping these fields of study, we can observe that the largest contributions come from Psychology (8276 publications), followed by Paediatrics (3226), Behavioural Sciences (3163), and Medicine (3129). Psychiatry and Neurosciences Neurology also play a crucial role, with 2956 and 2903 publications, respectively. Other significant fields addressing this research topic include developmental psychology, Autism-related studies, Language Development, Audiology Speech Language Pathology, Neuroscience, Rehabilitation, Educational Research, Social Sciences, Genetics, Linguistics, and Cognitive Psychology. Additionally, contributions from Arts and Humanities, Health Professions, Cognition, Clinical Psychology, Biochemistry, Genetics and Molecular Biology, Nursing, Computer Science, and Multidisciplinary fields provide a more diverse perspective on the subject. This distribution of research across various disciplines demonstrates the interdisciplinary nature of language acquisition in ASD, emphasizing the importance of integrating knowledge from different fields to enhance our understanding and support individuals with autism more effectively.

**Figure 5:**
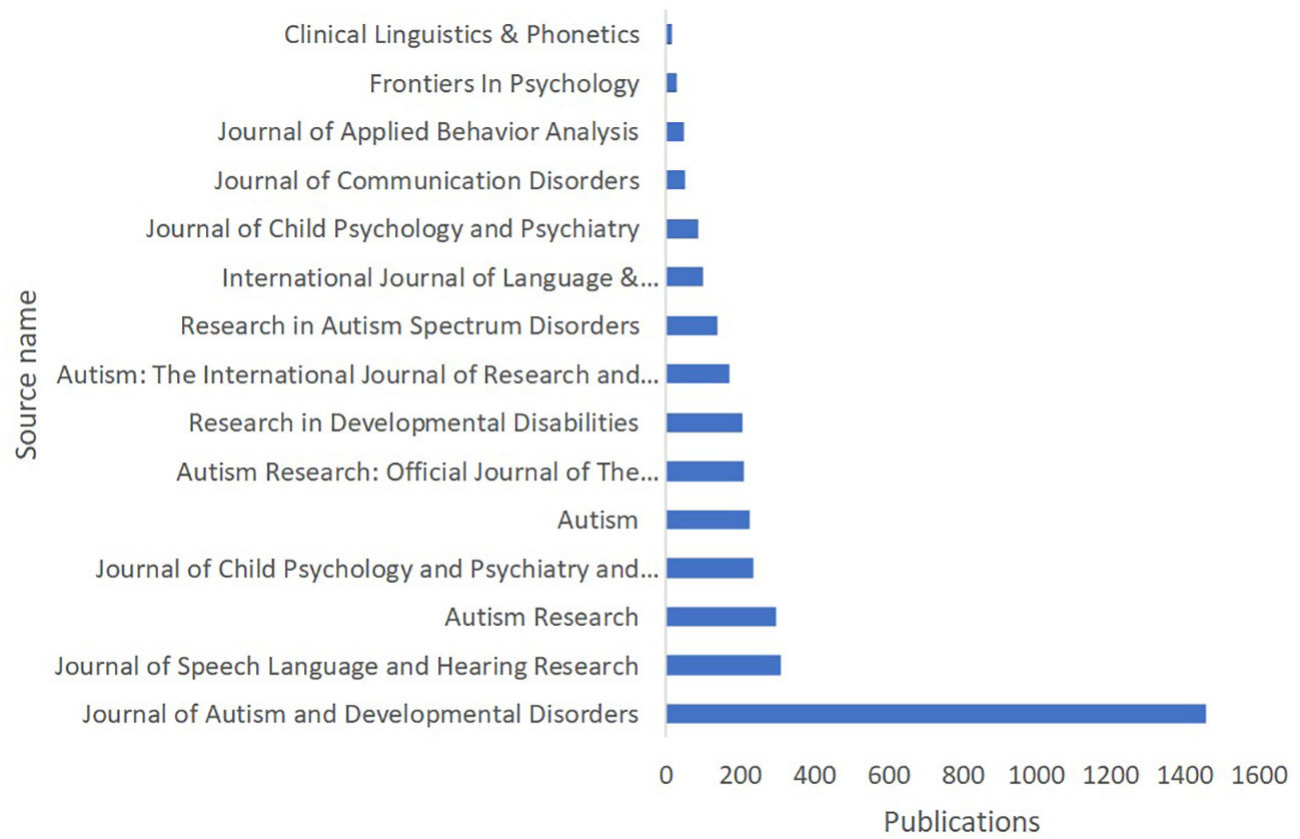
Language Acquisition in ASD: Knowledge Production by Source.

The data presented in **Figure 5** reveals the top publishing journals and sources in the research area of language acquisition in ASD, based on the total number of publications across three databases: Scopus, WoS, and Lens. The Journal of Autism and Developmental Disorders leads the field with a substantial 1458 publications, demonstrating its prominence as a key source for research in this area. The Journal of Speech Language and Hearing Research (310 publications) and Autism Research (299 publications) also contribute significantly to the body of knowledge. Other important sources include the Journal of Child Psychology and Psychiatry and Allied Disciplines (237 publications), Autism (226 publications), Autism Research: Official Journal of The International Society for Autism Research (211 publications), Research in Developmental Disabilities (206 publications), Autism: The International Journal of Research and Practice (170 publications), and Research in Autism Spectrum Disorders (140 publications). Additional noteworthy sources include the International Journal of Language & Communication Disorders, Journal of Child Psychology and Psychiatry, Journal of Communication Disorders, Journal of Applied Behaviour Analysis, Frontiers in Psychology, and Clinical Linguistics & Phonetics. These findings highlight the importance of specialized journals in disseminating research on language acquisition in ASD, allowing researchers to access relevant, high-quality information to advance the understanding and support of individuals with autism.

**Figure 6:**
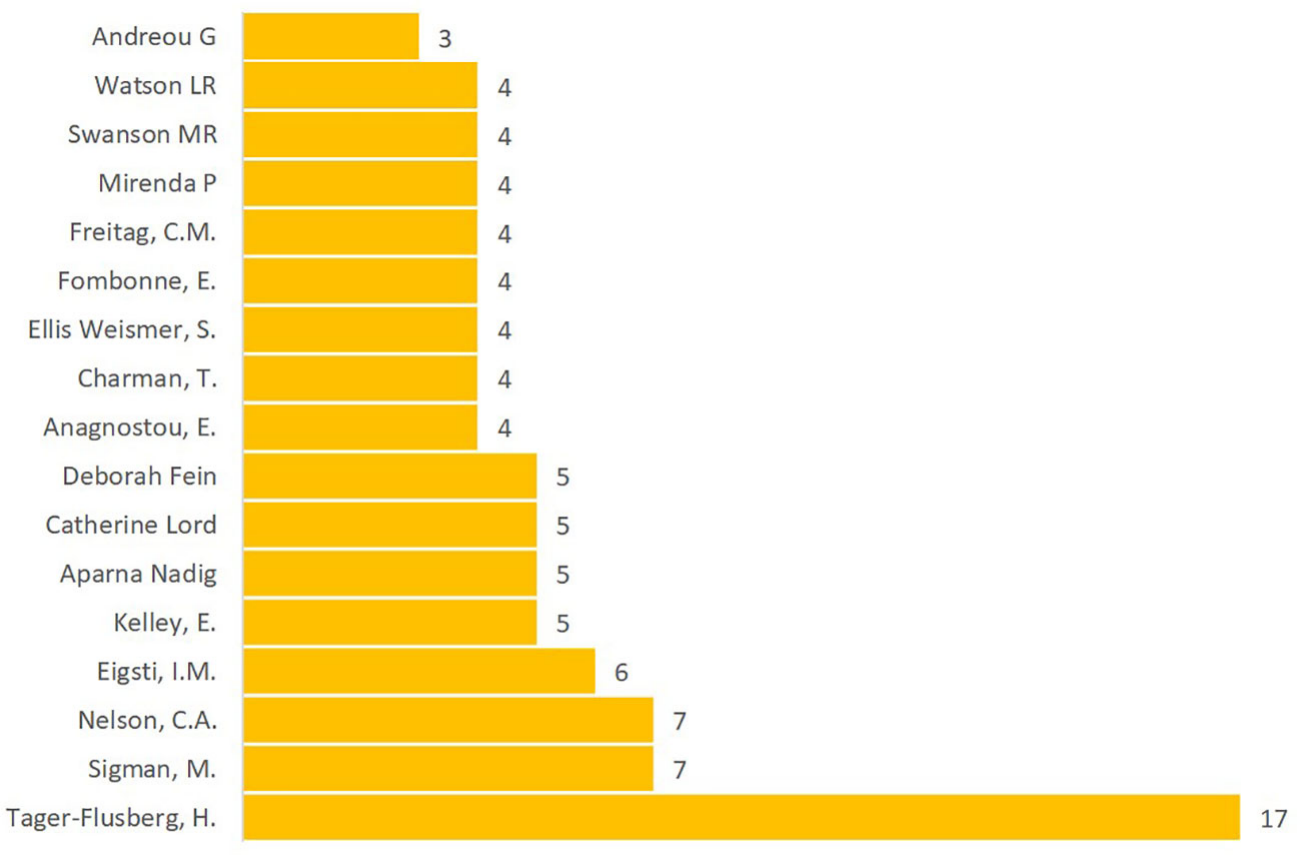
Language Acquisition and Development in ASD: Knowledge Production by Authors.

The examination of top authors contributing to research in language acquisition and development in ASD reveals a notable group of experts driving advancements in this field. The top ten authors, shown in **Figure 6**, were identified from three databases, Scopus, WoS, and Lens, and duplicates were removed, assigning the highest value for each author with duplicate entries. Tager-Flusberg (e.g., 46) stands out with 17 publications, showcasing her significant influence on the field. Other prominent authors include Sigman (e.g., 47)and Nelson (e.g., 48), each with 7 publications, followed by Eigsti (e.g., 49) with 6 publications. Researchers such as Kelley (e.g., 49) Nadig (e.g., 50), Lord (e.g., 51), and Fein (e.g., 52) have each made substantial contributions with 5 publications. A group of authors, including Anagnostou (e.g., 16), Charman (e.g., 53), Weismer (e.g., 54), Fombonne (e.g., 55), Freitag (e.g., 56), Mirenda (e.g., 12), Swanson (e.g., 9), and Watson (e.g., 20), have also had a notable impact, each with 4 publications. Lastly, Andreou (e.g., 10) has contributed with 3 publications. These top authors demonstrate a strong commitment to advancing our understanding of language acquisition and development in ASD. Their collective expertise and dedication have significantly shaped the research landscape in this area, providing valuable insights that inform the design of targeted interventions and guide future investigations in language development for individuals with ASD.

### 3.3 Emerging interests and clusters related to language acquisition in ASD

**Figure 7:**
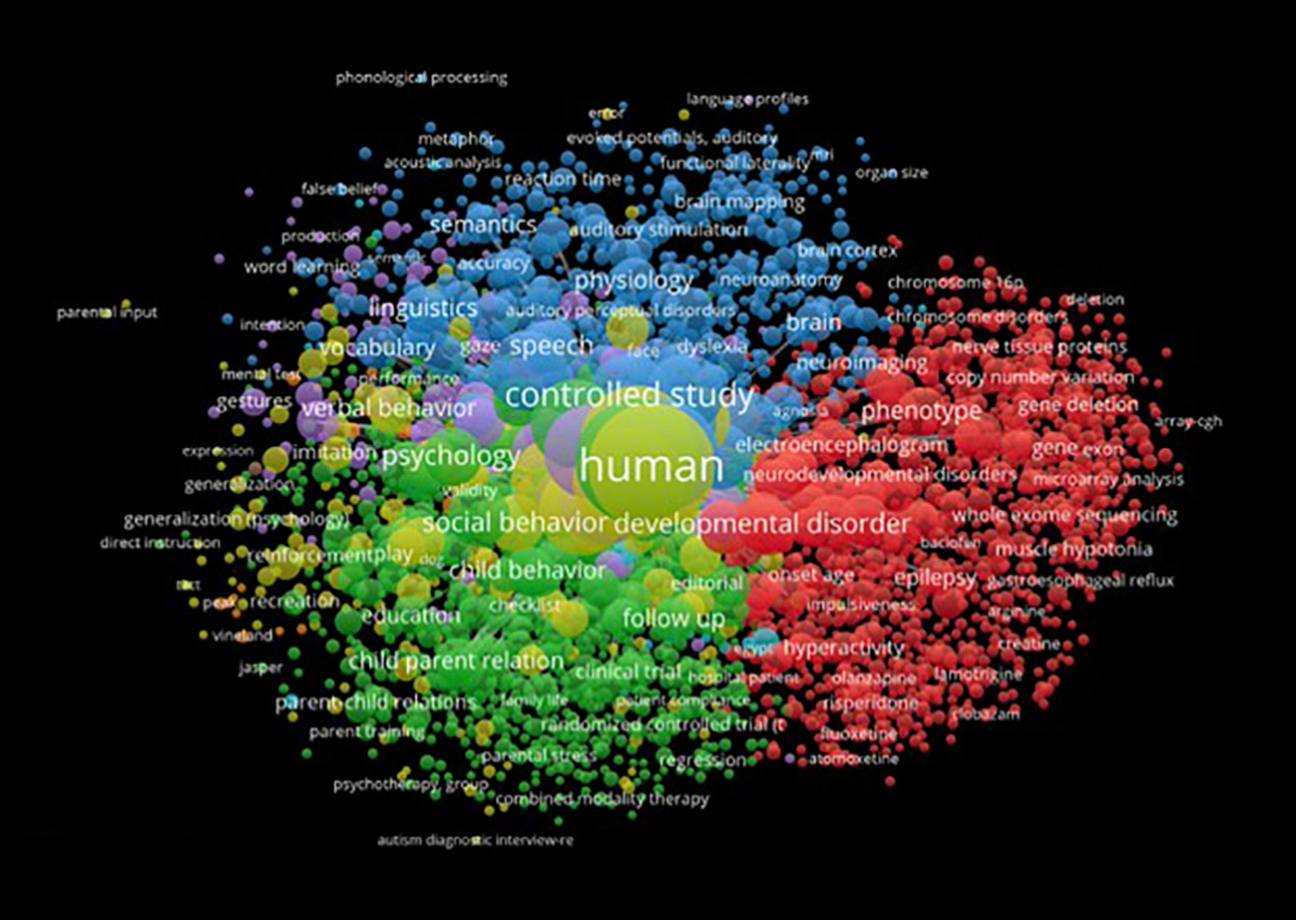
Network Visualization for Keywords Co-occurrence of Language Acquisition in ASD.

The network visualization in **Figure 7**, which displays a comprehensive map derived from merged data of Scopus, WoS, and Lens, reveals several implications for the study of language acquisition in relation to ASD. Out of 16,178 keywords, 2749 met the threshold of a minimum five-time frequency for inclusion, with nine distinct clusters represented by different colours. Another visualization demonstrating the top 100 co-occurred keywords is generated with the highest five clusters 1–5.

Clusters focusing on genetic aspects (Cluster 1), language ability and autism (Cluster 2), and age-related factors in autism (Cluster 3) suggest that language acquisition in ASD involves a complex interplay of genetic, developmental, and cognitive factors. This highlights the need for an interdisciplinary approach in understanding the diverse language-related challenges faced by individuals with ASD. The presence of clusters emphasizing language development (Cluster 5), language skills (Cluster 7), and language delay (Cluster 8) underscores the importance of early intervention strategies tailored to the specific needs of children with ASD. This could help mitigate language delays and improve long-term outcomes. Additionally, the clusters exploring hearing impairment and autism (Cluster 6) and IQ (Cluster 9) imply that language acquisition in ASD is influenced by various co-occurring conditions and cognitive abilities. Researchers and clinicians should be cautious in generalizing language development patterns and should consider individual differences when designing interventions.

In short, the visualization highlights the multifaceted nature of language acquisition in relation to ASD, emphasizing the need for interdisciplinary research and personalized intervention strategies to support the diverse language development needs of individuals with ASD.

**Figure 8:**
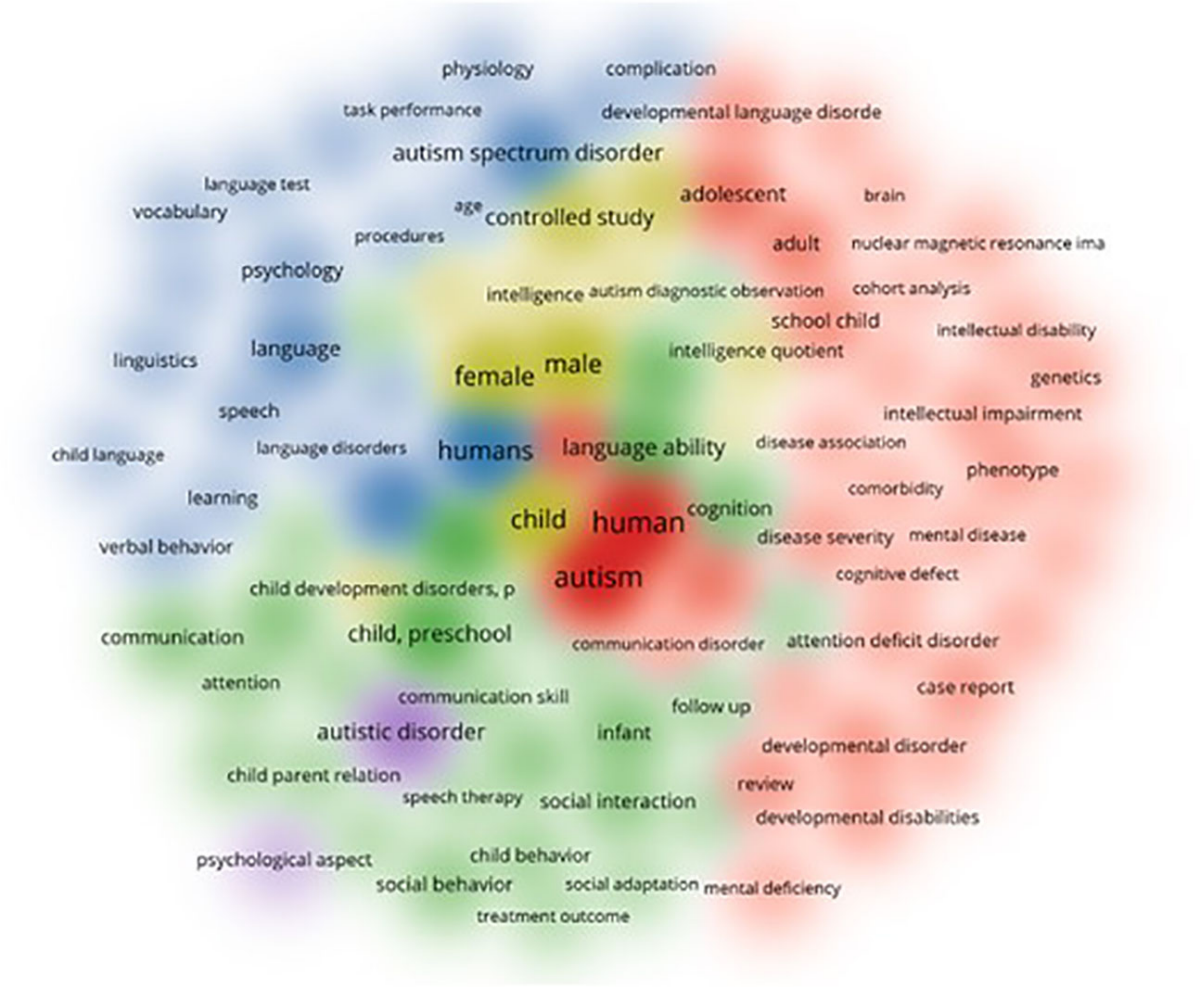
Density Visualization for Major Keywords Co-occurrence of Language Acquisition in ASD.

The density visualization in **Figure 8** presents another insightful map, showcasing the top 100 co-occurred keywords within the highest five clusters, numbered 1 through 5. This detailed representation allows for a closer examination of the most prevalent themes and relationships among these dominant clusters, enabling a better understanding of the core topics and their interconnectedness within the research landscape.

By focusing on the top 100 co-occurred keywords, the visualization highlights the key aspects and trends in each of the five clusters. For instance, it may reveal the most influential factors in genetic aspects of autism (Cluster 1), the major themes in language ability and autism (Cluster 2), and the most prominent age-related factors in autism (Cluster 3). Furthermore, it can shed light on the primary concerns in autistic disorder and related conditions (Cluster 4) and the crucial elements of language development (Cluster 5).

This targeted visualization not only provides a more in-depth understanding of the main topics within these five clusters but also enables researchers to identify potential areas of collaboration and interdisciplinary research. By exploring the interconnections between the keywords and their respective clusters, researchers can better comprehend how various factors intersect and influence one another, leading to enhanced knowledge synthesis and the development of innovative research approaches. In sum, the top 100 co-occurred keywords visualization serves as a valuable tool for uncovering the most salient aspects of these five highly influential clusters within the field.

The analysis of highly cited documents in the field of language acquisition in ASD, drawn from Scopus, WoS, and Lens, offers valuable insights into the impact and significance of these studies. The top 10 highly cited documents from each database, shown in **Table 3**, were compiled, duplicates removed, resulting in a final list of 16 unique documents. The high citation count of these documents can be attributed to their specific strengths or attributes, which have resonated with the research community and contributed to advancing the understanding of language development in individuals with ASD.

1. Longitudinal studies, such as those focusing on joint attention and language development in autistic children or comparing language acquisition in autistic and Down syndrome children, provide valuable insights into developmental trajectories and highlight the importance of early intervention strategies.
2. Studies examining cognitive and language skills in various populations, such as adults with autism and children with different developmental disabilities, offer a comprehensive understanding of the relationship between cognitive abilities and language development, informing targeted interventions.
3. Benchmarking studies, like the one defining spoken language benchmarks for young children with ASD, help establish clear goals and standards for assessing and monitoring progress in language development.
4. Investigating early predictors, such as gaze behaviour and affect at 6 months, offers insights into early indicators of language development and potential risk factors for ASD, enabling earlier identification and intervention.
5. Studies exploring the impact of specific interventions, like secretin administration, on social and language skills contribute to the development of novel treatment approaches for individuals with ASD.
6. Developmental reviews, such as the one on language acquisition in ASD, provide a comprehensive synthesis of existing knowledge, highlighting research gaps, and guiding future investigations.
7. Assessing language development with standardized measures, like the MacArthur Communicative Development Inventory, ensures reliable and valid evaluations of language abilities, informing evidence-based practice.
8. Studies examining the role of parent behaviours and child characteristics as predictors of language change in ASD emphasize the importance of considering these factors in both research and intervention design.
9. Investigations on the relationship between nonverbal communication, play, and language development in autistic children offer insights into the complex interactions between various developmental domains, informing holistic intervention approaches.
10. Comparing receptive and expressive language abilities in preschoolers with ASD highlights specific areas of impairment, guiding the development of targeted language interventions.
11. Focusing on specific subpopulations within the ASD spectrum, such as adolescent boys with Asperger syndrome, helps identify unique language and social skill profiles, informing tailored interventions.
12. Studies on the use of computer-assisted technologies (CAT) to enhance language development in children with ASD demonstrate the potential of technology-based interventions, encouraging further exploration and innovation in this area.

**Table 3:** Top cited documents of language acquisition in ASD from Scopus, WoS and Lens.

No.	Source title	Number of Citations	Citation
1	A longitudinal study of joint attention and language development in autistic children	946	(57)
2	Cognitive and language skills in autistic, mentally retarded, and normal children	354	(47)
3	Language acquisition in autism spectrum disorders: a developmental review	346	(49)
4	Measuring early language development in preschool children with autism spectrum disorder using the MacArthur communicative development inventory (infant form)	324	(53)
5	The autistic child: language development through behavior modification	310	(58)
6	Gaze behavior and affect at 6 months: predicting clinical outcomes and language development in typically developing infants and infants at risk for autism	305	(59)
7	Defining spoken language benchmarks and selecting measures of expressive language development for young children with autism spectrum disorders	297	(60)
8	A longitudinal study of language acquisition in autistic and down syndrome children.	266	(46)
9	Modeling longitudinal change in the language abilities of children with autism: parent behaviors and child characteristics as predictors of change	258	(61)
10	Social and language skills in adolescent boys with Asperger syndrome	248	(62)
11	Use of computer-assisted technologies (CAT) to enhance social, communicative, and language development in children with autism spectrum disorders	246	(48)
12	Improved social and language skills after secretin administration in patients with autistic spectrum disorders.	240	(63)
13	Preschoolers with autism show greater impairment in receptive compared with expressive language abilities	188	(64)
14	Predictors of language acquisition in preschool children with autism spectrum disorders	161	(51)
15	Nonverbal-communication and play correlates of language-development in autistic-children	148	(65)
16	Cognitive and language skills in adults with autism: a 40-year follow-up	145	(66)

These highly cited documents, with their specific strengths and attributes, have significantly influenced research and practice in the field of language acquisition and development in ASD. By drawing on the insights provided by these studies, researchers and practitioners can design more effective interventions and continue to advance our understanding of language development in individuals with ASD.

**Figure 9:**
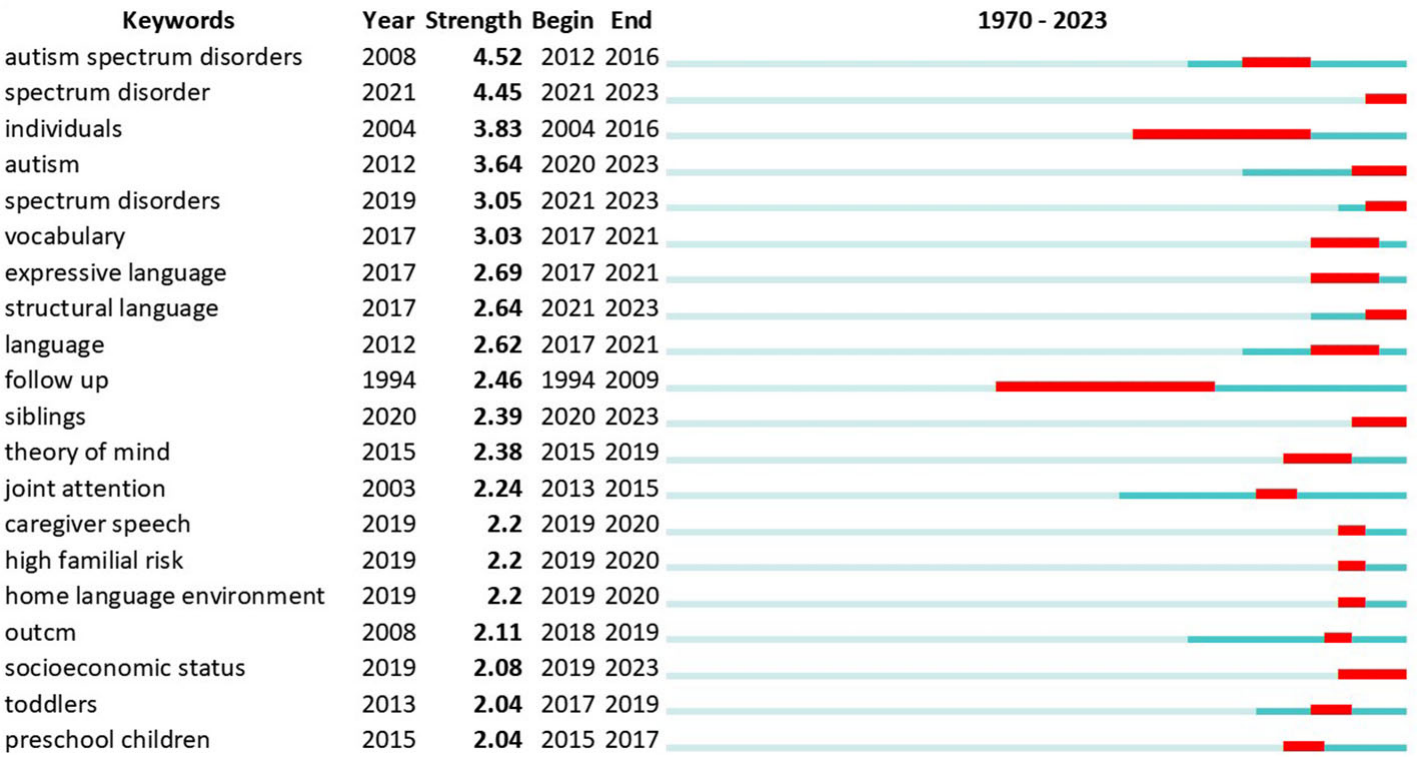
Top 20 Keywords with the Strongest Citation Bursts.

In the field of language acquisition in ASD, CiteSpace analysis on merged data from Scopus and WoS show the top 20 keywords with the strongest citation bursts (**Figure 9**). “Spectrum disorder” (2021–2023) and “autism spectrum disorders” (2012–2016) display the strongest citation bursts, suggesting a growing interest in understanding ASD as a spectrum, rather than a singular condition. The keyword with the longest burst duration is “individuals,” spanning from 2004 to 2016, underlining the importance of considering individual differences in ASD research. Notably, “vocabulary,” “expressive language,” and “structural language” emerged as important keywords, pointing to a focus on understanding and improving language abilities in individuals with ASD. Additionally, keywords like “siblings,” “theory of mind,” “joint attention,” and “caregiver speech” emphasize the significance of social and family factors in language development for ASD populations. These findings have implications for future research, highlighting the need for a comprehensive approach to studying ASD language acquisition, including the role of individual differences, family and social contexts, and the expanding understanding of ASD as a spectrum.

Cluster analysis is a valuable technique for uncovering patterns and relationships within large datasets. In the context of language acquisition in ASD, a CiteSpace analysis of merged data from Scopus and WoS has identified the top 10 clusters, each with unique implications for the main topic (**Table 4**). The Silhouette value, a measure used to evaluate the quality of clustering results, can provide further insights into the cohesion and separation of these clusters. A higher Silhouette value (closer to 1) indicates better clustering quality, while a lower value (closer to −1) indicates poor clustering quality.

1. Cluster 0 (Silhouette value: 0.776) - “autism spectrum disorder” with “fine motor gesture” and “usage pattern”: This cluster with a relatively high Silhouette value highlights the relationship between fine motor gestures and language usage patterns in individuals with ASD, indicating a need to explore motor skills and their impact on language development. The significant citing article of this cluster is by Joseph et al. (67).
2. Cluster 1 (Silhouette value: 0.739) - “autism spectrum disorder” with “expressive language skill” and “maternal gesture use”: This cluster, which has a moderately high Silhouette value, emphasizes the role of expressive language skills and maternal gesture use in language development for children with ASD, suggesting that parental communication strategies may influence language acquisition. The significant citing article of this cluster is by Brignell et al. (68).
3. Cluster 2 (Silhouette value: 0.789) - “autism spectrum disorder” with “online parent training module” and “prelinguistic predictor”: This cluster, with a relatively high Silhouette value, explores the effectiveness of online parent training modules for improving prelinguistic predictors of language outcomes in children with ASD, highlighting the potential of technology-based interventions. The significant citing article of this cluster is by Gabis et al. (69).
4. Cluster 3 (Silhouette value: 0.883) - “autism spectrum disorder” with “atypical language development” and “core language skills matter”: This cluster, which has a high Silhouette value, focuses on atypical language development in ASD and the importance of core language skills, suggesting a need for targeted interventions to address these foundational abilities. The significant citing article of this cluster is by Zoccante et al. (70).
5. Cluster 4 (Silhouette value: 0.784) - “Language development” with “natural language” and “syntactic complexity”: This cluster, with a relatively high Silhouette value, investigates the role of natural language input and syntactic complexity in language development for individuals with ASD, emphasizing the importance of considering linguistic features in research and interventions. The significant citing article of this cluster is by Menn et al. (17).
6. Cluster 5 (Silhouette value: 0.881) - “autism spectrum disorder” with “procedural memory network” and “prelinguistic predictor”: This cluster, which has a high Silhouette value, examines the relationship between procedural memory networks and prelinguistic predictors in ASD, pointing to the potential influence of cognitive processes on language development. The significant citing article of this cluster is by Chuthapisith et al. (71).
7. Cluster 6 (Silhouette value: 0.847) - “autism spectrum disorder” with “caregiver speech” and “prelinguistic predictor”: This cluster, with a high Silhouette value, highlights the role of caregiver speech in shaping prelinguistic predictors of language outcomes in children with ASD, reinforcing the importance of family and social factors in language development. The significant citing article of this cluster is by Swanson et al. (9).
8. Cluster 7 (Silhouette value: 0.86) - “Language ability” with “high functioning adult” and “signalling pathway gene”: This cluster, which has a high Silhouette value, explores the genetic underpinnings of language abilities in high-functioning adults with ASD, suggesting a potential biological basis for observed language differences. The significant citing article of this cluster is by MacDonald-Pregent et al. (50).
9. Cluster 8 (Silhouette value: 0.876) - “autism spectrum disorder” with “language comprehension” and “autism spectrum disorder”: This cluster, with a high Silhouette value, focuses on language comprehension difficulties in individuals with ASD, underscoring the need for interventions targeting this specific aspect of language development. The significant citing article of this cluster is by Brignell et al. (68).
10. Cluster 9 (Silhouette value: 0.808) - “Adaptive behaviour” with “adaptive behaviour” and “autism spectrum disorder”: This cluster, with a relatively high Silhouette value, examines the connection between adaptive behaviour and language development in ASD, highlighting the importance of considering broader functional skills in relation to language abilities. The significant citing article of this cluster is by Park et al. (72).

**Table 4:** Summary of the largest clusters of language acquisition in ASD.

ClusterID	Size	Silhouette	Label (LSI)	Label (LLR)	Label (MI)	Average Year
0	50	0.776	autism spectrum disorder	fine motor gesture	usage pattern	2012
1	49	0.739	autism spectrum disorder	expressive language skill	maternal gesture use	2014
2	47	0.789	autism spectrum disorder	online parent training module	prelinguistic predictor	2013
3	44	0.883	autism spectrum disorder	atypical language development	core language skills matter	2010
4	40	0.784	language development	natural language	syntactic complexity	2016
5	37	0.881	autism spectrum disorder	procedural memory network	prelinguistic predictor	2014
6	28	0.847	autism spectrum disorder	caregiver speech	prelinguistic predictor	2015
7	27	0.86	language ability	high functioning adult	signalling pathway gene	2016
8	23	0.876	autism spectrum disorder	language comprehension	autism spectrum disorder	2010
9	22	0.808	adaptive behaviour	adaptive behaviour	autism spectrum disorder	2016

In summary, these top 10 clusters generated from the CiteSpace analysis, along with their respective Silhouette values, provide valuable insights into various aspects of language acquisition in ASD. The high Silhouette values indicate that these clusters are well-separated and cohesive, suggesting that they capture meaningful patterns. By focusing on these clusters, researchers can delve deeper into the interconnected factors, such as motor skills, parental communication strategies, cognitive processes, and genetics, that contribute to language development in individuals with ASD. This comprehensive understanding can ultimately inform the creation of more targeted and effective interventions to support language acquisition in this population.

## 4 Discussion

The current scientometric review provides a comprehensive overview of the research landscape in language acquisition in ASD. This analysis has identified several primary trends, key contributors, and potential knowledge gaps in the field.

The primary trends in ASD language acquisition research include topics such as motor skills, parental communication strategies, cognitive processes, and genetics, which align with the findings of Eigsti et al. (49). The methods used in these studies are diverse, including both quantitative and qualitative approaches, echoing the approach advocated by Fombonne (73). Furthermore, the publication geography is widespread, with most of the research being produced by countries with well-established research infrastructure and resources, such as the United States and the United Kingdom. This distribution of research production underscores the importance of fostering international collaboration, as suggested by Park et al. (72).

The key contributors to ASD language development research are primarily academic institutions and researchers with a strong commitment to advancing understanding in this field. For instance, Tager-Flusberg has published extensively on this topic (46, 60), as have Sigman and Lord (47, 51). Their success can be attributed to their rigorous methodologies, innovative approaches, and dedication to enhancing knowledge and intervention strategies for individuals with ASD.

The emerging interests in ASD language acquisition research revolve around the use of technology in interventions, the role of individual and genetic differences, and the impact of social and family contexts on language development. For example, the use of computer-assisted technologies (CAT) to enhance social, communicative, and language development in children with ASD is a promising avenue for future research (48). Moreover, individual, and genetic differences in language ability among individuals with ASD have been increasingly recognized, underscoring the need for personalized approaches in research and interventions (14, 50).

However, some potential knowledge gaps have also been identified. For instance, the impact of linguistic features in natural language input on language development in ASD is an area that warrants further investigation (17). Additionally, the influence of broader functional skills, such as adaptive behaviour, on language abilities in ASD is not yet fully understood and requires more in-depth exploration (14).

Future studies can address these gaps by designing research that incorporates these emerging interests and unexplored areas. For instance, studies could investigate the impact of different types of natural language input on language development in ASD or examine the relationship between adaptive behaviour and language abilities in this population. Furthermore, researchers could continue to explore the use of technology in language interventions for individuals with ASD, as well as consider the influence of individual and genetic differences in their study designs. This multifaceted approach would not only enrich our understanding of language acquisition in ASD but would also inform the creation of more targeted and effective interventions to support these individuals and their families.

To sum up, this scientometric review has highlighted the complexity and diversity of research in ASD language acquisition, emphasizing the need for a comprehensive, interdisciplinary approach in future studies. By addressing the identified trends, key contributors, emerging interests, and knowledge gaps, researchers can continue advancing our understanding of ASD and developing more effective support strategies for those with autism and their families.

### 4.1 Emerging interests and addressing knowledge gaps in autism spectrum disorder language acquisition research

The analysis reveals several research hotspots and areas of interest. For instance, the importance of considering motor skills, parental communication strategies, cognitive processes, and genetics in language development for individuals with ASD is highlighted (Clusters 0, 1, 2, 3, 5, and 7). This aligns with the findings of Eigsti et al. (74) which emphasize the need to consider these factors in ASD research.

Moreover, the analysis shows an increasing interest in exploring the role of natural language input and syntactic complexity in language development for individuals with ASD (Cluster 4). This area of research could potentially enhance our understanding of how various linguistic features influence language development in ASD, thereby informing the design of more effective language interventions.

Furthermore, the review underscores the importance of considering broader functional skills, such as adaptive behaviour, in relation to language abilities in ASD (Cluster 9). This suggests that future studies should take a more holistic approach to understanding and supporting language development in ASD by considering a range of functional abilities and skills.

However, despite these emerging interests, some potential knowledge gaps remain. One such gap is the need for more research on the efficacy and generalizability of machine learning approaches in detecting ASD from language samples. While Wawer et al. (27) have made significant strides in this area, more research is needed to validate these findings in larger and more diverse populations.

Another gap highlighted by the review is the limited knowledge on the most effective strategies parents can employ to support language development in children with ASD. Several studies have highlighted the role of parents in this process (9, 20, 21), but there is a need for more research on how these strategies can be tailored to individual children’s needs.

In conclusion, the findings of this scientometric review underscore the need for a comprehensive, interdisciplinary approach to studying language acquisition in ASD. Future studies should aim to address the identified knowledge gaps and continue to explore the emerging interests in this field. Doing so will not only enrich our understanding of language development in ASD but also inform the creation of more effective and tailored interventions to support individuals with ASD and their families.

## 5 Conclusion

This scientometric review aimed at providing a detailed analysis of the research landscape in language development in ASD exploring the trends, patterns, and knowledge gaps. The methods employed in this review consisted of a systematic search of three major databases: Scopus (5026 documents), Web of Science (WoS; 4570 documents), and Lens (3235 documents). The analysis included bibliometric indicators of knowledge production size by year, country, university, source, subject area, author, and citation. Scientometric indicators included the burst detection, silhouette, clusters, citation, and the co-occurrence of keywords. The analysis showed that clusters focusing on various aspects of language development in ASD, such as motor skills, parental communication strategies, cognitive processes, and genetics. Key clusters showed the interrelationship between fine motor gestures and language usage patterns, the role of expressive language skills and maternal gesture use, and the effectiveness of online parent training modules for improving prelinguistic predictors. Some other clusters highlighted the importance of core language skills, the role of natural language input and syntactic complexity, and the genetic underpinnings of language abilities in high-functioning adults with ASD. In conclusion, this scientometric review highlighted the top 10 clusters and their respective Silhouette values, providing valuable insights into language acquisition in ASD. Based on our scientometric review of the literature we conducted, our detailed analyses presented from various studies provides valuable direction to future research on language acquisition of ASD to design effective interventions.

## Author contributions

MA: Conceptualization, Funding acquisition, Project administration, Supervision, Writing – original draft. AA: Data curation, Formal analysis, Methodology, Resources, Software, Validation, Visualization, Writing – original draft, Writing – review & editing. FQ: Conceptualization, Funding acquisition, Investigation, Project administration, Resources, Writing – original draft.
